# Gossip

**Published:** 1863-07

**Authors:** 


### Art. XI.—GOSSIP.

The Medical Council has brought to its close another session of laborious trifling; filling the pages of our unlucky weekly contemporaries with closely-printed reports, resolutions, and amendments, and doing nothing with the greatest possible amount of verbosity and display. As a matter of principle and plain public duty, we have already urged the admission of reporters to the meetings; but we cannot suggest to the profession that we, or any one, can lose anything by the fact that these gentlemen are still pertinaciously excluded.

The way in which the Medical Council deals with a question of importance is as follows:—A member of Council proposes a motion, to which, after it has been duly seconded, several amendments are proposed also. The amendments are first negatived, and in due course the original motion is negatived likewise. After each division somebody demands that the names of the majority and minority should be entered upon the minutes; Drs. A., B., and C. are therefore "written down" upon one side or the other, and the question at issue remains precisely in its original condition.

Not only in the ambition of being "written down" does the Council sustain the character of a corporate Dogberry, but also, and even more completely, in its dealings with "vagrom men," represented for the nonce by recalcitrant examining bodies.

"This is your charge; you shall comprehend all vagrom men. You are to bid any man stand, in the prince's name."

"How if a' will not stand?"

"Why, then, take no note of him, but let him go; and presently call the rest of the watch together, and thank God you are rid of a knave. . . . . You are to call at all the alehouses, and bid those that are drunk get them to bed."

"How if they will not?"

"Why, then, let them alone till they are sober. If they make you not then the better answer, you may say they are not the men you took them for."

"Well, sir?"

"If you meet a thief, you may suspect him, by virtue of your office, to be no true man; and, for such kind of men, the less you meddle or make with them, why, the more is for your honesty."

"If we know him to be a thief, shall we not lay hands on him?"

"Truly, by your office you may; but, I think, they that touch pitch will be defiled. The most peaceable way for you, if you do take a thief, is to let him show himself what he is, and steal out of your company."*

So far the parallel is complete; but the Council have still to master the principle, that "for the watch to babble and talk is not tolerable and not to be endured." The Royal College of Surgeons have steadily refused adherence to the Council scheme of medical education, and the Council have neither had the courage to maintain their original position, nor the wisdom to recede from it with dignity. The College of Surgeons has taken the trouble to assign reasons for its decision, but we should suppose that no examining body will take this trouble for the future. At the first breath of opposition the Medical Convocation has collapsed like a punctured wind-bag.

---

* *Much Ado about Nothing*, act iii. scene 3.

The question of medical education has called forth a remarkable memorial from the medical staff of the Devon and Exeter Hospital, and a somewhat similar document from the Exeter Eye Infirmary. It appears that the unsettled state of the whole matter, and the promulgation of regulations that were at first expected to be enforced, have interfered with the supply of pupils and apprentices to these and similar institutions. The medical officers, accustomed to derive considerable fees from these pupils, and the governors of hospitals, accustomed to get a great deal of dressing and dispensing done by them without payment, are very naturally dissatisfied; and the memorials before us are intended to show that valuable educational power is likely to be wholly wasted. The staff of the Devon and Exeter Hospital, with sarcasm not the less bitter for being apparently unintentional, impute to the Council ignorance of the habits of life and modes of practice of the great body of practitioners, and explain this ignorance by the "high position, social and professional," of the councillors themselves. The memorialists proceed, in effect, to urge a return to the old system of medical education, as most calculated to form the "practical and ready man;" and they think that an early initiation into medical and surgical routine is more valuable than the general education that must be sacrificed in order to attain it. They refer to the expense of the new system, as a further objection to its general adoption; and conclude with a paragraph which, being interpreted, means that if a student aspire to be a councillor by-and-bye, he ought certainly to receive a decent education; but that, if his ambition be less aspiring, he may do very well without one. If he can spread blisters and draw teeth at fifteen, he will have no need of book-learning.

In former numbers of this journal we have so recently considered this view of the educational question, that we need not now repeat ourselves. That the general status of the profession is lower than it ought to be, because the education of its members has been sacrificed to routine and cheapness, is, it seems to us, a mere truism; and it is equally a truism to say that a more complete and scientific education would bring into the profession men more able to appreciate the complicated problems of life and disease, more removed from the considerations that influence the petty tradesman, and better furnished with the pecuniary means of waiting a reasonable time for success. Our present low standard has in no way operated more prejudicially than by its cheapness. Youths have been apprenticed to the neighbouring parish doctor by parents scarcely able to maintain them for three years at a hospital. As soon as qualified they have been left to fight their own way; and their struggles and competitions, quite unavoidable under the circumstances, have tended to lower the tone of the whole medical body, besides having exercised a most hurtful influence on medical remuneration. "Clubs" at three shillings a member, including middle-class tradesmen, and union appointments paying an average of three-farthings or three-halfpence a case, are only rendered possible by the action of the cheap education that finds favour in the West.

In the suggested waste of Educational power there is perhaps some truth. But we consider the problem before us to be, not how quickly and cheaply a youth may be prepared to discharge, without disgraceful failure, the routine duties of general practice, but how he can be most thoroughly fitted for the varied and onerous responsibilities of medical and surgical life. We apprehend that the use of country hospitals as training schools might with great advantage follow after the metropolitan course of study.

As at present constituted, country hospitals are rarely calculated to lay a good foundation to the medical character. They are seldom kept up to the high-water mark of science; and they are usually centres and hot-beds of professional vices. The members of the medical staff are almost always too much rivals in private to be cordial colleagues in their public practice; they very generally owe their positions to birth, or marriage, or sect, or some influence altogether apart from fitness; and they find it necessary to maintain an attitude of passive resistance against the possible encroachments of their brethren without. Improvements in surgery are commonly resisted by obstructive colleagues, and petty jealousies reign supreme. During this present year we have visited a large county hospital, with four surgeons, distant more than a hundred miles from London, in which excision of the knee-joint has never been performed. The surgeons say they do not like "new fangles," and they continue to amputate through the thigh, as their fathers did before them. We have visited another, of almost equal importance, in which no member of the staff had ever seen a laryngoscope; and we have visited a county eye infirmary of great repute, in which the ophthalmoscope was scarcely used at all, and never used efficiently, and in which no test-types or lenses were ever employed. Asthenopia was treated by solutions of caustic, and diagnosis of internal disease was left to the guess-work of ten years ago. Now these cases, although extreme, are not exceptional, and they indicate a moral atmosphere very bad for a young student. Practitioners must expect to meet with many of their brethren who are wanting in professional zeal; but the generous rivalry of metropolitan schools and teachers is a stimulus admirably calculated to awaken zeal in pupils who have not been already subjected to unfavourable influences. Young men who go from a good school to a London hospital will regard the lessons of the latter from a different point of view from that of those who for four or five years have seen country magnates sacrifice the welfare of patients, and fall behind the progress of science, in order to snub professional rivals, and to indulge professional jealousies.

The Pharmacopœia is promised in the course of the coming autumn. As if fearful that the results of their labours would not duly impress the public mind, the committee have placed upon record a statement of the number of meetings and attendance by which these results have been obtained.

On a former occasion we inquired the meaning of "infamous conduct in a professional respect." The question will be answered in time by a method of exclusion. By degrees we shall learn what is not infamous. One Mr. Jordan, who has been struck off the list of members of the Royal College of Surgeons, on account of his connexion with an exhibition of obscene waxworks, is still retained upon the register. The Council have been compelled, of course, to erase his surgical qualification; but they do not take any independent action in the matter.

A variety of routine business has also been transacted, of no particular importance to anybody. The exclusion of reporters, carried by a considerable majority formerly, rested, this year, upon the casting vote of the president, and will doubtless be negatived in the next session. We shall then be able to judge, better than now, the reasons for the ludicrous uselessness of the Council. That it is too large for an executive, and too small for a deliberative body, is at once apparent. When the speeches of the various councillors are reported we may perhaps discover that the interests of rival boards and universities are so exactly balanced as to produce a dead lock; and that an efficient Council can only be obtained by a more fair and complete method of professional representation.

The Governors of Bethlehem Hospital are desirous of emulating the notoriety of their brethren of St. Thomas's Hospital, and up to the present time their success has been most praiseworthy. Indeed, since the publication of our last number, the former have contrived to excite public odium to an extent almost equal to that educed by the more protracted self-willed folly of the latter. Early in the year, the Governors of St. Thomas's submitted to the Governors of Bethlehem proposals for obtaining the site of the latter hospital. St. Thomas's offered to purchase a fitting site for and build a new Bethlehem at a cost not exceeding 150,000*l.*, or to pay Bethlehem that sum for the present site and hospital. After dallying with this handsome proffer for some time, Bethlehem, to the amazement of all who had not reflected on the probability that its Governors might possibly be fabricated of the same material as those of St. Thomas's, suddenly closed the negotiation; refusing to concur in any plan which should have for its first object an agreement as to the probable cost of a new Bethlehem, and resolving that no proposal for an exchange of sites could be entertained which was not based on an understanding that the whole cost of the removal of Bethlehem should be borne by St. Thomas's! That is to say, that St. Thomas's was to be permitted to carry out its proposition on the condition only of paying such unbounded costs as the whim or fancy of the Governors of Bethlehem might dictate! This was the notion of a business negotiation at Bethlehem. Here, then, much to the mortification of St. Thomas's and the disgrace of Bethlehem, the matter rested. St. Thomas's could not well complain, for she had been paid out in her own coin. Bethlehem had elected to do what St. Thomas's had at first done, that is, to leave the real question at issue in the removal of the hospital, to wit, the most legitimate and best method of securing its objects, entirely out of consideration. The public fortunately think these objects of greater importance than the pitiful conceit of the Governors, and Bethlehem is not to be allowed, no more than St. Thomas's, to settle matters according to her own peculiar prejudices, in utter forgetfulness of the unfortunates she was created to give succour to. The Commissioners of Lunacy have interfered, and addressed the following letter to the Secretary of State for the Home Department:—

"Office of Commissioners in Lunacy,
19, Whitehall-place, June 3.

"Sir—The Commissioners in Lunacy have watched with much interest the proceedings and discussions which have for some time past been before the public in reference to the question of the purchase of Bethlehem Hospital by the Governors of St. Thomas's, and the removal of the first-named institution to a healthy and suitable locality in the neighbourhood of London. It was the earnest hope of the Commissioners that the protracted negotiations which have so long occupied the attention of the promoters and wellwishers of both those important and wealthy charities would ere this have eventuated in an arrangement so desirable, and recommended by such obvious considerations of the great benefits which would thence be derived by the unfortunate objects of the respective institutions.

"It is, however, with Bethlehem Hospital and its insane inmates that the Commissioners are specially concerned, and it is with the view, ere it be too late, of urging upon the Governors the expediency and duty of availing themselves of the present favourable opportunity to give the insane the advantages of pure air and cheerful scenery, so essential to health, mental and bodily, that the Commissioners are induced to address to Secretary Sir George Grey the present communication

"It has for many years been the opinion of the Board that the site of Bethlehem Hospital, as respects its limited extent and situation in the centre of a dense and rapidly increasing population, is most unsuited to the due medical care and treatment of the insane, for whose sole benefit the administration of its ample property and income is intrusted to the Governors. Outdoor exercise and recreation, and freedom from disturbance and observation, so indispensable to the proper treatment of insanity, especially in its earlier stages, require an ample extent of grounds and gardens within the boundaries of the institution. In all these respects Bethlehem Hospital is essentially defective, and the Commissioners are unwilling to believe that the Governors can have finally closed the door against the offer of an eligible site in the country, and within a convenient distance of London, of which they have it in their power now to avail themselves.

"The views above expressed have always been entertained by the Commissioners, and were embodied in a communication to Mr. Secretary Walpole as far back as November, 1858; wherein, with reference to a collateral question, the Commissioners adverted to the consistency of the opinion conveyed with the reasons they had 'given for suggesting the removal of Bethlehem Hospital from its present populous locality into the country.'

"The observations above made have been confined to the question of the site; a most important objection to Bethlehem Hospital, as a place for the treatment and cure of insanity, remains to be noticed—viz., the unfitness, according to modern opinions, of the building in respect to its construction and arrangements. The general aspect of the hospital, externally and internally, notwithstanding the efforts made within the last few years to enliven the long corridors and day-rooms, cannot but exercise a depressing influence upon the inmates, whose means of outdoor exercise are so limited and inadequate. The Commissioners, in the case of asylums for pauper lunatics, would never sanction plans upon the principle of Bethlehem Hospital.

"The new hospital, which the Commissioners still trust will be built in the country, will of course be constructed upon a plan embodying all the improvements suggested by modern experience and the advanced state of science.

"The large funds at the disposal of the Governors confer upon them almost unprecedented means of improving the care and treatment of the insane, and consequently impose upon them, in an especial manner, the duty and responsibility of applying those funds in the manner best calculated to promote and extend the objects and benefits of the institution, which cannot be done upon the present site. The opportunity now offered to remove the hospital to a suitable rural locality may never occur again.

"It will be for Sir George Grey to consider in what way effect can be best given to the views which the Commissioners have thus endeavoured to bring under special notice, and in the greatness and importance of which they doubt not he will concur.

"I am, &c.,

"W. C. Spring Rice, Secretary.

"H. Waddington, Esq., Home-office."

This letter was forwarded by the Secretary of State to the Governors of Bethlehem, and was discussed by them in a meeting held on the 23rd June. The Governors were very irate at the interference, and said many foolish things singularly condemnatory of the management of the hospital. The meeting, however, adjourned without coming to a decision as to what answer should be made to the Home Secretary, but a committee was appointed for the purpose of discussing the matter calmly.

In the meantime the medical officers of St. Thomas's have addressed a memorial to the Governors of that hospital on the question of site. They name two as of first-rate excellence:—the site of Bethlehem, and a site about to be created opposite the Houses of Parliament, on the banks of the river, by the Metropolitan Board of Works. The latter site they unanimously conclude to be the best of the two, and earnestly recommend it to the choice of the Governors.

An effort is about to be made to convert the labour now lying dead in the cotton manufacturing districts to some good use. It is proposed, as far as possible, to find employment for the unoccupied operatives on some public works. To ascertain the practicability of, and facilitate this object, the Government instructed Mr. Robert Rawlinson, civil engineer, in May last, to visit the principal towns of Lancashire, and report on the works which might with advantage be instituted there. He was directed to inspect the towns and their suburbs; to ascertain the character of the drainage, and the quantity and quality of the water supplied; the condition of the streets; the provision for parks and pleasure-grounds for the recreation of the inhabitants; and the general sanitary arrangements of each locality. Mr. Rawlinson was also to form an opinion as to what works of utility, profit, or ornament, were capable of being executed; to make, as far as possible, an estimate in each case of the cost of such works; to lay his suggestions upon these points before the local authorities, and explain to them the beneficial results which might be obtained by carrying out the works, both in the improvement of the towns and in the employment of the distressed operatives, at a fair rate of wages, which latter was "the immediate and pressing object for which works of this nature were mainly to be undertaken."

Mr. Rawlinson, in a preliminary report, expresses himself favourably of the scheme. There are no towns in Lancashire which are not susceptible of great sanitary improvement, and upon which money might not be expended with much profit, present and prospective, to the population. Ashton-under-Lyne, Mr. Rawlinson informs us, would do well to disburse 40,000*l.* on main sewers, additional water supply, road and street improvements, and a public park. Blackburn wants about twelve miles of main sewers, and twelve miles of streets completed, at a cost of some 70,000*l.* Oldham could very usefully spend 100,000*l.* on sewers, street improvements, and a public park. Rochdale would benefit by an expenditure of 70,000*l.*, also on sewers, streets, and a park. The operatives must be supported, and a sound economy points out that in supporting them it is best not to let their labour lie idle, but to use it for the public good. We trust that the proposition to employ this labour on public works will prove every way feasible.

The recent alarming outbreak of small-pox in the metropolis, it is to be hoped will lead to some more efficient legislative arrangements being made to secure trustworthy vaccination. There can be no doubt that the frequently recurring outbreaks of small-pox to which this country is subjected, are entirely due to the scandalously imperfect character of the provisions for public vaccination. The inquiries into this subject instituted at the instance of the Medical Officer of the Privy Council, and published in his third and fourth annual reports, demonstrate this fact. As an illustration of the imperfect system of public vaccination now in vogue in this country, it may be mentioned that of 694 districts inspected, only 64 were found where the public vaccinator professed to follow regularly the plan of vaccination prescribed by his contract. The whole system of public vaccination requires to be revised and removed from the baneful control of parish authorities.

